# Miscellaneous Medical Intelligence

**Published:** 1849-07

**Authors:** 


### ARTICLE VII.

### MISCELLANEOUS MEDICAL INTELLIGENCE.

The late destructive fire in St. Louis has prevented the issue of the St. Louis Med. and Surgical Journal, the office of publication having been destroyed. So soon as other arrangements can be made, the editors will resume publication.

Dr. Henry J. Bigelow has been appointed to the Chair of Surgery in Harvard University, in place of Prof. Hayward, resigned.

Dr. B. F. Silliman, Jr., has been elected to the Professorship of Chemistry in the University of Louisville, in place of Prof. Yandell, who has been transferred to the Chair of Physiology and Pathology in the same institution.

Sulphuric acid is said to be a test of the purity of Cod-liver oil. Mixed in the proportion of four parts of the latter with one of the former, a rich violet hue is obtained, passing gradually into a dirty brown. This characteristic is possessed by no other oil, animal or vegetable.

Prof. Bartlett of Transylvania University has accepted the chair of the Principles and Practice of Medicine in the University of Louisville, rendered vacant by the resignation of Prof. Drake.

Dr. Hunt has been appointed to the Chair of Physiology and Pathology in the University of Louisiana, to fill the vacancy occasioned by the death of the lamented Prof. Harrison.

Woods' Quarterly Abstract of the Medical Sciences has been discontinued for want of promptness on the part of subscribers.

A new Medical Journal is to be issued at Lexington, Kentucky, under the editorial supervision of Prof. E. L. Dudley.

There are five hundred and twenty practitioners of medicine in New Hampshire.

